# Lysosomal Storage Disorders: Molecular Basis and Therapeutic Approaches

**DOI:** 10.3390/biom11070964

**Published:** 2021-06-30

**Authors:** Enrico Moro

**Affiliations:** Department of Molecular Medicine, University of Padova, via U.Bassi 58/b, 35131 Padova, Italy; enrico.moro.1@unipd.it

**Keywords:** lysosomal storage disorders, biomarkers, gene therapy

Lysosomal storage disorders (LSDs) are a group of 60 rare inherited diseases characterized by a heterogeneous spectrum of clinical symptoms, ranging from severe intellectual disabilities, cardiac abnormalities, visceromegaly, and bone deformities to slowly progressive muscle weakness, respiratory insufficiency, eye defects (corneal clouding and retinal degeneration), and skin alterations [1]. Pioneering biochemical studies between the early 1970s and 1990s attributed the pathogenesis of LSDs to a disrupted catabolic function of lysosomal enzymes and consequent primary lysosomal substrate storage [2]. However, in the past two decades, a wealth of published research expanded this classical view to a more complex scenario, whereby multiple primary defects produced by lysosomal enzyme deficiency concur, leading to a range of cellular abnormalities, including oxidative stress, mitochondrial alterations, cell signaling defects, and calcium dyshomeostasis [3,4]. This Special Issue covered an overview of the current knowledge regarding the pathogenesis of different lysosomal diseases and their therapeutic perspectives. In their work, Hampe and colleagues provided an exhaustive description of therapeutic approaches for Mucopolysaccharidosis type I, including enzyme replacement therapy (ERT) and hematopoietic stem cell transplantation (HSCT). Throughout the paper, the authors claimed that both treatments do not provide full recovery from primary symptoms and suggested that early diagnosis is critical for correct therapeutic management [5]. Meena and Raben discussed similar findings in their review on Pompe disease, covering novel aspects of disease pathogenesis, including the role of autophagic impairment in glycogen storage and therapeutic advances in the field of ERT. In their detailed overview of the different alpha-glucosidase (GAA) formulations developed over the years, the authors pointed out that significant, but still limited, clinical improvements have been achieved in affected patients [6]. Regarding the same topic, Murray described his findings on glycogen-containing carbohydrates masked by an unknown protein derived from the recombinant GAA (rhGAA)-dependent glycogen breakdown outside of the lysosome and the cell. The author proposes the use of these new detected terminal degradation products of rhGAA in the serum as biomarkers for follow-up and treatment protocols [7]. Gragnaniello and collaborators presented their long-term experience on a wide newborn screening for Fabry disease and proposed lyso-Gb3 as a useful biomarker for diagnostic and follow-up protocols [8]. Kok and colleagues collected an exhaustive overview of Fabry disease pathogenesis and treatment, considering the role of neutralizing antibodies against recombinant enzymes, which are responsible for the relapse in plasma lysoGb3 levels after several years of ERT in affected patients. The authors stressed the prompt need to develop alternative therapeutic strategies, of which α-1,4-Galactosyltransferase (A4GALT) inhibitors represent a quite promising approach [9]. In research of the same disease, Ivanova and colleagues described their findings on the role of clathrin-mediated endocytosis of recombinant alpha-galactosidase A (rh-α-Gal A) in different experimental cellular models. Interestingly, they provided limited but clear evidence that rh-α-Gal A uptake was responsible of autophagy induction in their experimental models [10]. Impaired intracellular trafficking was also evoked in the paper by Barnes and colleagues who presented their intriguing data on TGFβ1 missorting and increased sortilin levels in experimental models of mucolipidosis type II (MLII) [11]. In the field of sphingolipidoses, Limgala and Goker-Alpan provided a preliminary description of the measured plasma levels of secreted biomarkers, including osteopontin (OPN), osteoprotegerin (OPG), and chemokine (C-C motif) ligand 18 (CCL18) and percentages of T and B-lymphocytes in Gaucher patients under ERT and SRT [12]. Indeed, Srikanth and Feldman reported a very interesting study on the extracellular Dickkopf-1 (Dkk1)-mediated downregulation of the canonical Wnt pathway in an induced-pluripotent stem cell model of neuronopathic Gaucher disease [13]. Three other elegant reviews also contributed to this Special Issue: the work of Pinto e Vairo and colleagues reported a summarizing overview on the relevance of precision medicine in the field of lysosomal storage disorders [14], while Massaro and colleagues included their comprehensive summary of the currently available and developing gene therapy approaches and clinical trials in the management of lysosomal diseases [15]. Gleason and colleagues reported an excellent collection of data related to the significance of exosomes in the context of lysosomal disorders pathogenesis, but the authors also emphasized the clinical application of exosomes as therapeutic delivery vehicles [16]. In one additional review, Manzoli and colleagues raised an important and puzzling question related to the potential relevance of investigating the axonal guidance-related aspects in lysosomal disorders. The authors provided an extensive list of axon guidance diseases exhibiting clinical features resembling those of lysosomal disorders [17]. Tonazzini and colleagues described through the Twicher (TWI) mouse, the most used model of Krabbe disease, the onset of visual impairment, reduced contrast sensitivity, and neuropathological signs, including astrogliosis and reduced myelination in the early life stages [18]. Finally, De Pasquale and collaborators reported the application of a label-free quantitative proteomic approach in a mucopolysaccharidosis type IIIb mouse model, which enabled the classification of three major clusters of proteins dysregulated in the diseased brain [19].

Altogether, the articles of this Special Issue have broadened our concepts in the field of lysosomal storage disorders, offering a reference cue for the pathogenic aspects and evolving therapeutic approaches related to these rare diseases.
